# A unifying molecular mechanism underlying the association of *CARD14* alleles with autoinflammatory and T-cell mediated skin disorders

**DOI:** 10.1186/1546-0096-13-S1-O50

**Published:** 2015-09-28

**Authors:** D Berki, S-E Choon, AD Burden, C Griffiths, C Smith, J Barker, F Capon

**Affiliations:** 1 King's College London, London, UK; 2 Hospital Sultanah Aminah, Johor Bahru, Malaysia; 3 University of Glasgow, Glasgow, UK; 4 University of Manchester, Manchester, UK

## Introduction

The *CARD14* (Caspase Recruitment Family Member 14) locus encodes a scaffold protein that mediates NF-kB signalling in keratinocytes and is therefore crucial to the maintenance of skin immune homeostasis. In keeping with this notion, gain-of-function *CARD14* mutations have been observed in patients with plaque psoriasis and pityriasis rubra pilaris, two skin disorders mediated by abnormal T cell activation. More recently, a *CARD14* missense variant has been tentatively associated with generalised pustular psoriasis (GPP), an auto-inflammatory condition characterised by acute episodes of skin pustulation and systemic upset.

## Objectives

The aim of this study was to establish whether *CARD14* alleles are genuinely associated with GPP and to investigate the molecular mechanism underlying any effect on disease risk.

## Patients and methods

We investigated an extended case cohort (n=100) ascertained in Europe and East Asia. As all disease alleles described to date cluster to exons 3 and 4, we screened this mutation hotspot in all patients. We also sequenced the entire *CARD14* coding region in a subset of 16 individuals. We analysed population matched, control genotypes (n= 997) that were generated in-house or had been previously released by the 1000 Genomes Consortium. Finally, we investigated the accumulation of CARD14 oligomers by western blotting, following the transfection of HEK293 cells with wild-type or mutant cDNA constructs.

## Results

We found that a non-conservative p.Asp176His substitution was significantly associated with GPP in the Chinese and Japanese populations (combined *P*=0.0001; OR:5.3). Bioinformatics showed that this change had pathogenic potential and was likely to disrupt the coiled coil of CARD14. Importantly, our analysis predicted a similar effect for p.Glu138Ala and p.Leu156Pro, two disease alleles previously associated with psoriasis and pityriasis rubra pilaris. Since the coiled coil domain of CARD14 mediates protein oligomerization, we investigated the effects of the above mutations on the accumulation of CARD14 aggregates. We found that all three disease alleles caused spontaneous protein oligomerization.

## Conclusion

Given that CARD14 oligomerization is a pre-requisite for downstream signal transduction, our results indicate that disease alleles promote abnormal NF-kB signalling by causing constitutive protein aggregation. Thus, our work points to a unifying pathogenic mechanism underlying the effects of *CARD14* mutations on auto-inflammatory and T-cell mediated disorders.

